# Plaque removal by a novel prototype power toothbrush versus a manual toothbrush: A randomized, exploratory clinical study

**DOI:** 10.1002/cre2.556

**Published:** 2022-04-21

**Authors:** Paola Gomez‐Pereira, Alyson Axe, Andrew Butler, Jimmy Qaqish, Chhaju R. Goyal

**Affiliations:** ^1^ GSK Consumer Healthcare Weybridge Surrey UK; ^2^ All Sum Research Mississauga Ontario Canada

**Keywords:** gingivitis, oral hygiene, plaque, power toothbrush

## Abstract

**Objectives:**

This exploratory study investigated plaque removal with a prototype constant, low rotation speed Power Toothbrush (PTB) with two brushing actions: “Gumline” (head rotates in the horizontal axis) and “Interdental” (head rotates in the vertical axis). Gumline alone and “Combined” (Gumline + Interdental) modes were compared with a Reference PTB and a Reference Manual Toothbrush (MTB) after one brushing.

**Materials and Methods:**

Thirty‐nine participants were randomized to use each toothbrush once either in the sequence (A) Prototype PTB (in Gumline then Combined mode), (B) reference MTB, and (C) reference PTB or the sequence BAC. There was a minimum 3‐day washout between the use of each toothbrush. Plaque removal was measured using the Rustogi Modified Navy Dental Plaque Index (RMNPI) with change from baseline investigated using an analysis of covariance model. RMNPI scores were calculated on a “whole mouth” basis and along the gingival margin and at proximal sites only.

**Results:**

For the primary efficacy variable, a significant difference was found in favor of the prototype PTB in gumline mode versus the reference MTB for whole mouth plaque score (difference: −0.06; standard error: 0.014; 95% confidence interval [CI] −0.09 to −0.04; *p* < .0001). Similar significant differences were found in gingival margin and proximal areas (*p* < .0001). The prototype PTB in gumline mode removed significantly less plaque than the prototype PTB in combined mode and the reference PTB (*p* < .0001; whole mouth/gingival/proximal areas). The prototype PTB in combined mode removed significantly more plaque than the reference MTB (*p* < .0001; whole mouth/gingival/proximal areas) and the reference PTB for whole mouth (*p* = .0214) and gingival margin areas (*p* = .0010). The reference PTB also removed significantly more plaque than the reference MTB (*p* < .0001; whole mouth/gingival/proximal areas). All brushes were generally well‐tolerated.

**Conclusion:**

The prototype PTB design, providing two distinct cleaning modalities, can effectively remove plaque to a significantly higher degree than an MTB and a marketed PTB, depending on mode.

## 1 INTRODUCTION

Dental plaque is a diverse and organized community of tooth surface microorganisms. Via an ordered sequence of events, a matrix of polymers of host and bacterial origin coalesce to form the microbial biofilm. This process starts just minutes after toothbrushing with the formation of a protective saliva pellicle on the tooth surface (Samaranayake & Matsubara, 2017). Left undisturbed, the biofilm can develop into a more pathogenic composition that can eventually lead to periodontal disease (Harvey, 2017).

Bacteria that form biofilms in dental plaque are protected by the biofilm polymer matrix so are less susceptible to antimicrobial agents found in toothpaste or mouthwash (Hathroubi et al., 2017); therefore, mechanical removal methods, such as with a toothbrush, are an essential part of dental plaque control. There is a large body of evidence, including a Cochrane review, amounting to decades of research regarding the safety and efficacy of power toothbrushes (PTBs) (Ng et al., 2020; Wilder & Bray, 2016; Yaacob et al., 2014). These studies indicate that PTBs are safe to use and, compared to manual toothbrushes (MTB), remove significantly more dental plaque and significantly reduce gingivitis in the short (<3 months) and long term (>3 months) (F. A. Van der Weijden et al., 2011; Yaacob et al., 2014). PTB technology also has the potential to enhance motivation and compliance to achieve higher levels of dental plaque removal—for example, by the use of built‐in timers and quad pacers—and consequently improve the gingival condition (Delaurenti et al., 2017; Ng et al., 2020; Wilder & Bray, 2016).

PTBs were first introduced commercially in the 1950s (Ng et al., 2020; Yaacob et al., 2014); since then, there have been significant innovations improving PTBs with regard to aiding the user to carry out optimal daily oral hygiene, such as changes in frequency and amplitude of brushing (Starke et al., 2019). However, despite the relatively large adoption of PTBs in high‐income countries (study sponsor data on file), periodontal disease has a high prevalence in the adult populations of such (Nazir et al., 2020). With this in mind, new approaches are being developed to further improve plaque removal either by improving compliance (i.e., with the use of personalized/customized apps) or through novel technologies, such as refinements to PTB design and use of associated smartphone applications (Erbe et al., 2018, 2019; Wilder & Bray, 2016).

The PTB device tested in this exploratory clinical study is an early prototype with a novel technology proposed to offer superior cleaning at a constant and low speed of rotation. The prototype PTB operates in two different cleaning modes according to the axis of rotation of the head: “Gumline,” where the head rotates in the horizontal axis, and “Interdental,” where the head rotates in the vertical axis. These can be used one after the other for a “Combined” mode.

This study aimed to investigate plaque removal efficacy of the prototype PTB after a single brushing when used in Gumline or Combined modes compared to a marketed Reference PTB and an MTB. An investigation was carried out for the whole mouth and for gingival margin and proximal sites alone. Plaque levels were measured using the Rustogi Modified Navy Dental Plaque Index (RMNPI) (Rustogi et al., 1992), an index widely used in PTB studies that can show differentiation in dental plaque removal in the approximal tooth regions (Biesbrock et al., 2007; Goyal et al., 2009; Klukowska, Grender, Goyal, et al., 2012; Rosema et al., 2016; Sharma et al., 2011). The study also had a safety objective to evaluate the oral tolerance of the toothbrushes.

## 2 METHODS

This study was an exploratory, randomized, four‐treatment, three‐period, study in healthy, right‐handed MTB users. It was carried out at a Canadian Research facility in full compliance with the International Council for Harmonisation of Technical Requirements for Registration of Pharmaceuticals for Human Use, all applicable local good clinical practice regulations and participant privacy requirements, and the ethical principles outlined in the Declaration of Helsinki. The final study protocol, informed consent forms, and other information that required preapproval were reviewed and approved by an independent institutional review board (Veritas Investigational Review Board, Inc., Quebec, Canada: IRB Approval Number 2017‐GSK‐3‐IRB‐16333). Anonymized individual participant data and study documents can be requested for further research from www.clinicalstudydatarequest.com. ClinicalTrials.gov Identifier: NCT03809910.

### 2.1 Procedures

At the screening visit (Visit 1), participants aged 18–65 years gave their written informed consent before any study procedures taking place. Eligible participants underwent an oral soft tissue (OST) and oral hard tissue (OHT) examination. Study eligibility included being right‐handed, regularly using an MTB, and being in good general and dental health. Participants needed to have at least 20 permanent gradable teeth (restorative materials covering <25% of the graded tooth surface) and a mean RMNPI whole mouth plaque score of ≥0.6 at each of the study visits, before brushing (see below for scoring).

Exclusion criteria included smoking/chewing tobacco or E‐cigarette use; pregnancy; breastfeeding; any clinically significant and relevant abnormality in medical history or upon oral examination that could, in the examiner's opinion, affect study participation and intolerance/hypersensitivity to study materials. Further exclusions included: use of antibiotic treatment or chlorhexidine mouthwash within 2 weeks of screening; dental prophylaxis within 4 weeks of screening; undergone tooth bleaching/whitening within 8 weeks of screening; received orthodontic therapy or scaling or root planing within 3 months of screening, or/and been treated for periodontal disease within 12 months of screening. Specific dental exclusions included: restorations in a poor state of repair; high levels of extrinsic stain or calculus deposits that could interfere with plaque assessments; oral/perioral ulceration; orthodontic bands/appliances, extensive crowns, full/partial dentures, fixed retainers, tongue or lip piercings that could interfere with toothbrushing; active caries, excessive gingival recession, severe gingivitis or periodontitis that, in the examiner's opinion, could interfere with study participation.

Before study toothbrush use, participants underwent a prebrushing OST examination followed by plaque disclosing according to the disclosing solution manufacturer's instructions (Trace® solution; Young™ Innovations, Inc. Algonquin, IL, USA). This was followed by a prebrushing dental plaque assessment using the RMNPI (see below). All assessments were carried out by the same examiner throughout the study (CRG). Supervised training was provided so that participants could familiarize themselves with how the different modes and different PTBs operated. Eligible participants were provided with a fluoride toothpaste (Colgate® Cavity Protection Toothpaste; Colgate‐Palmolive Co.) and an MTB (Colgate Extra Clean®, soft bristles; Colgate‐Palmolive Co.) to use at home throughout the study period. Diary cards were used to note all brushing occasions. Participants were requested to not have any elective dental procedures, use any whitening treatment or carry out any interproximal cleaning during the study period.

Following the screening visit, participants attended three treatment visits (Visits 2, 3, and 4). Each visit was followed by a minimum 3‐day washout period during which participants brushed with the standard fluoride toothpaste and toothbrush. Before each site visit, participants abstained from all oral hygiene for at least 12 h. On study days, participants abstained from all food and drink (except water) or from chewing gum for at least 4 h before their scheduled visit.

**Figure 1:**
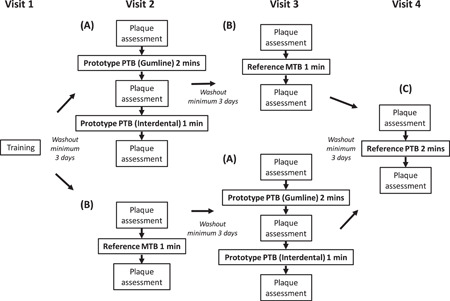
Study procedures

Eligible participants were randomized to the order in which they used the toothbrushes according to a schedule provided by an independent statistical organization (ABC/BAC design) (Figure 1). As the Reference PTB was a marketed, high‐end product, the user experience with it was expected to be inherently different than with the Prototype PTB, which did not have all the features of a finished product. As such, to minimize user bias, the Prototype PTB (A) was always used before the Reference PTB (C).

Conditions were (Figure 1): (A) Prototype PTB (head and handle), used for 2 min in Gumline mode, RMNPI assessment, then brushing for a further 1 min in Interdental mode, RMNPI assessment again; (B) Reference MTB as a negative control (Oral‐B® Indicator 1–2–3 toothbrush medium; Procter & Gamble Co.), used for 1 min with instructions to brush in their usual manner, then RMNPI assessment; (C) Reference PTB as a positive control (Oral‐B® Genius 8000 rechargeable PTB Handles with Oral‐B® Cross Action toothbrush head; Procter & Gamble Co.) used in “Daily Clean” mode (as per manufacturer's instructions) for 2 min, then RMNPI index assessment. Due to the nature of the study design, participant blinding was not possible.

At each study visit, a dental plaque was first disclosed and assessed using the RMNPI. Following this, a trained study site staff member applied 1.3 g (±0.1) of toothpaste to the assigned toothbrush head and demonstrated the correct use of the assigned toothbrush. Participants brushed accordingly, then plaque was assessed as before (Figure 1). An OST examination followed each brushing occasion.

At Visit 2, brushes in conditions (A) or (B) (according to schedule assignment) were used as instructed, followed by the washout period. At Visit 3, conditions were swapped such that each participant completed condition (A) or (B) in their assigned order. Following another washout period, all participants completed condition (C) during Visit 4 (Figure 1).

All examinations and assessments were carried out by a single dental examiner. At Visits 2, 3, and 4, repeatability data on two participants (one prebrushing and one postbrushing) were generated for plaque assessment from replicate examinations, at least 10 min apart, on the same participant. If deemed necessary by the examiner, plaque could be redisclosed if the dye had faded.

Adverse events (AEs) and incidents were collected throughout the study. As this was the first use in the human study, incidents/device failures were monitored during and after all uses of the Prototype PTB.

### 2.2 RMNPI

**Figure 2:**
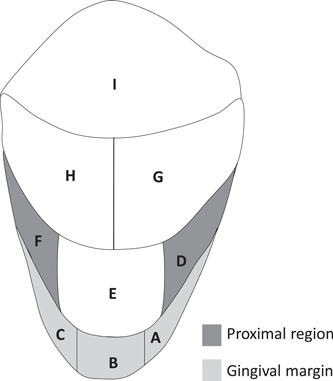
Rustogi Modified Navy Dental Plaque Index. Adapted from Rustogi et al. (1992)

The RMNPI scores dental plaque in proximal tooth areas and at the gum line as well as over the total tooth buccal/lingual surface (Rustogi et al., 1992) (Figure 2). A plaque was evaluated as either present or absent (1 or 0) on each of nine areas of the buccal and nine areas of the lingual tooth surfaces. Dental plaque was assessed on all teeth excluding third molars, crowns, and surfaces with cervical restorations, for a maximum of 28 eligible teeth (504 gradable sites), with a minimum number of 18 teeth (324 gradable sites). Participants' RMNPI scores were calculated on a whole mouth basis (Sites A–I), along the gingival margin (Sites A, B, and C), and at proximal sites (D and F).

### 2.3 Efficacy variables

The primary objective was to investigate and compare plaque removal efficacy of the Prototype PTB used in the Gumline mode versus the Reference MTB after a single brushing, as measured by the RMNPI whole mouth score. Change from prebrushing in whole mouth RMNPI score for all teeth was derived from the individual sites for each tooth first before calculating the average change in whole mouth score. Secondary objectives were to investigate and compare as above using RMNPI gingival margin and proximal scores as well as to compare the Prototype PTB in Combined mode (Gumline followed by Interproximal modes) versus the reference MTB; the Prototype PTB in Gumline and in Combined modes versus the Reference PTB; and the Prototype PTB in Gumline versus Combined modes, all as measured by the RMNPI.

### 2.4 Analysis

A sufficient number of participants were to be screened to randomize approximately 35 participants and ensure 30 completed the study. With 30 participants, it was possible to detect a mean treatment difference of 0.025 (standard deviation [*SD*] = 0.048) between the Prototype PTB in Gumline mode versus the MTB in the pre–post brushing RMNPI whole mouth score after a single‐use (primary objective) with 80% power and a 5% significance level. The estimated standard deviation (SD) was obtained from studies with a similar design (Sharma et al., 2011).

The modified intent‐to‐treat (mITT) population was used for the analysis of plaque score, which included all randomized participants who used a study product at least once and provided at least one postbrushing assessment of efficacy. The safety population included all eligible participants who had received supervised PTB training.

For the primary comparison, change from pre‐brushing in the whole mouth RMNPI score was analyzed using analysis of covariance (ANCOVA) with the product group and sequence as fixed effects, participant as a random effect, and two baseline terms as covariates: (i) participant‐level baseline score calculated as mean prebrushing score across all visits within a participant, and (ii) period level baseline minus participant level baseline. The adjusted mean change from pre‐brushing scores, standard error (SE), 95% confidence interval (CI), and *p* values for the Prototype PTB Gumline and Reference MTB product groups are presented. All statistical tests of hypothesis were two‐sided and employed a level of significance of *α* = 0.05. Assumption of residual normality and homogeneity of variance in the ANCOVA analysis were tested and were considered satisfactory. All secondary comparisons were analyzed in a similar manner to the primary.

Repeatability data to investigate intraexaminer variability were generated for RMNPI from replicate examinations compared to the original assessments. The repeat assessments were not used in any efficacy analyses. The first and repeat plaque assessments on each tooth site were cross‐tabulated and a weighted Kappa coefficient (*κ*) using the Fleiss–Cohen method of weighting. Analyses were conducted on the Repeatability Population, comprising all participants who had a repeat clinical assessment of efficacy at any visit.

## 3 RESULTS

**Figure 3:**
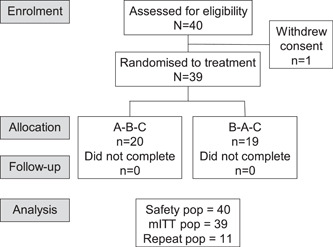
Study Flow. mITT, modified intent‐to‐treat

This study took place between July 8, 2019 and August 9, 2019. A total of 40 participants were screened for eligibility and took part in training (Safety population); 39 were randomized and completed treatment (Figure 3). Slightly more participants were male (*n* = 20; 51.3%) and participants were either of White/European (*n* = 16; 41.0%), Asian (*n* = 13; 33.4%), African American/African (*n* = 9; 23.1%), or mixed (*n* = 1; 2.6%) heritage. Mean age was 42.2 years (SD 14.75; range 18–63 years).

### 3.1 Primary efficacy

**Figure 4:**
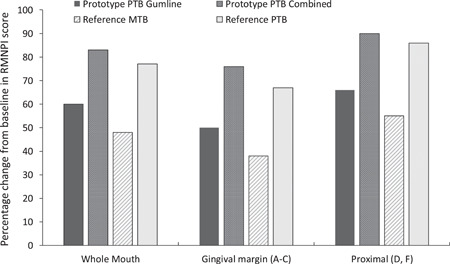
Percentage change from baseline in Rustogi Modified Navy Dental Plaque Index score (modified intent‐to‐treat population)

The primary efficacy variable was changed from prebrushing to postbrushing RMNPI whole mouth score (Sites A–I) (Table 1; Figure 4). Prebrushing whole mouth RMNPI score (mean ± SE) was the same for the Prototype PTB in Gumline mode (0.65 ± 0.007) and Reference MTB (0.65 ± 0.007). Postbrushing, whole mouth RMNPI score reduced significantly (*p* < .0001) with the use of either toothbrush (Table 1). Between‐group comparison of change from prebrushing in the whole mouth RMNPI score (adjusted mean difference ± *SE*) significantly favored the Prototype PTB in Gumline mode versus the Reference MTB (−0.06 ± 0.014) (95% CI −0.09 to −0.04; *p* < .0001) (Table 2).

**Table 1:** Rustogi Modified Navy Dental Plaque Index score change from baseline (modified intent‐to‐treat population)

	Prototype PTB Gumline	Prototype PTB Combined	Reference MTB	Reference PTB
	Whole mouth mean (SE)			
Prebrushing	0.65 (0.007)	0.65 (0.007)	0.65 (0.007)	0.65 (0.006)
Postbrushing	0.26 (0.018)	0.11 (0.012)	0.34 (0.016)	0.15 (0.009)
Adj mean change (SE) [95% CI]	−0.38 (0.012)	−0.54 (0.012)	−0.32 (0.012)	−0.50 (0.012)
*p* value	[−0.41 to −0.36] <.0001	[−0.56 to −0.51] <.0001	[−0.34 to −0.30] <.0001	[−0.53 to −0.48] <.0001
	Gingival margin (A–C sites) mean (SE)			
Prebrushing	1.00 (0.000)	1.00 (0.000)	1.00 (0.000)	1.00 (0.000)
Postbrushing	0.50 (0.025)	0.24 (0.023)	0.62 (0.027)	0.33 (0.016)
Adj mean change (SE) [95% CI]	−0.50 (0.023)	−0.76 (0.023)	−0.38 (0.023)	−0.67 (0.023)
*p* value	[−0.54 to −0.45] <.0001	[−0.81 to −0.71] <.0001	[−0.42 to −0.33] <.0001	[−0.72 to −0.63] <.0001
	Proximal (D, F sites) mean (SE)			
Prebrushing	1.00 (0.000)	1.00 (0.000)	1.00 (0.000)	1.00 (0.000)
Postbrushing	0.34 (0.028)	0.10 (0.017)	0.45 (0.022)	0.14 (0.015)
Adj mean change (SE) [95% CI]	−0.66 (0.021)	−0.90 (0.022)	−0.55 (0.021)	−0.86 (0.022)
*p* value	[−0.70 to −0.62] <.0001	[−0.94 to −0.85] <.0001	[−0.06 to −0.51] <.0001	[−0.90 to −0.82] <.0001

*Note*: Adj mean: Adjusted mean change from baseline using ANCOVA model.

Abbreviations: ANCOVA, analysis of covariance; CI, confidence interval; MTB, manual toothbrush; PTB, power toothbrush; SE, standard error.

**Table 2:** Comparison between groups in adjusted mean change from baseline (modified intent‐to‐treat population)

Group		Whole mouth	Gingival margin (A–C)	Proximal (D, F)
		Adjusted mean difference (standard error) [95% confidence interval] *p* value		
Prototype PTB Gumline Vs	Reference PTB	0.12 (0.014)	0.18 (0.025)	0.20 (0.025)
		[0.09 to 0.15] <.0001	[−0.13 to 0.23] <.0001	[0.15 to 0.25] <.0001
	Prototype PTB Comb	0.15 (0.014)	0.26 (0.025)	0.23 (0.025)
		[0.13 to 0.18] <.0001	[0.21 to 0.31] <.0001	[0.18 to 0.28] <.0001
	Reference MTB	**−0.06 (.014)**	−0.12 (0.025)	−0.11 (0.025)
		**[−0.09 to −0.04] <.0001** a	[−0.17 to −0.07] <.0001	[−0.16 to −0.06] <.0001
Prototype PTB Combined Vs	Reference MTB	−0.22 (0.014)	−0.38 (0.025)	−0.34 (0.025)
		[−0.25 to −0.19] <.0001	[−0.43 to −0.33] <.0001	[−0.39 to −0.29] <.0001
	Reference PTB	−0.03 (0.014)	−0.09 (0.025)	−0.04 (0.026)
		[−0.06 to −0.00] .0214	[−0.14 to −0.04] .0010	[−0.09 to 0.01] .1652
Reference MTB Vs	Reference PTB	0.19 (0.014)	0.30 (0.025)	0.31 (0.025)
		[0.16 to 0.21] <.0001	[0.25 to 0.35] <.0001	[0.26 to 0.36] <.0001

*Note*: Adjusted mean difference: First product minus second product where a negative difference favors first product and positive difference favors the second.

Abbreviations: MTB, manual toothbrush; PTB, power toothbrush.

^a^ Primary comparison.

### 3.2 Secondary efficacy

#### 3.2.1 Whole mouth (Sites A–I), all but primary variable

Prebrushing mean (±SE) whole mouth RMNPI score for the Reference PTB 0.65 (±0.006) was comparable with the Prototype PTB and Reference MTB (above). Postbrushing, scores reduced significantly with the use of all toothbrushes (within‐group *p* < .0001 for all) but the reduction was greatest with the Prototype PTB in Combined mode (Table 1; Figure 4). Between‐group comparisons (Table 2; Figure 4) of change from prebrushing scores significantly favored the Prototype PTB Combined mode versus the Prototype PTB Gumline mode, the Reference MTB, and the Reference PTB (all *p* < .0001). The Reference PTB was significantly favored over the Prototype PTB Gumline mode (*p* < .0001) and the Reference MTB (*p* = .0214).

#### 3.2.2 Gingival margin sites (A–C)

Prebrushing mean (±SE) gingival margin sites score was the same for all toothbrush groups: 1.00 (±0.000). Postbrushing scores reduced significantly with all toothbrushes (within‐group *p* < .0001); the reduction was greatest for the Prototype PTB in Combined mode (Table 1; Figure 4). Between‐group comparisons of change from prebrushing scores (Table 2; Figure 4) significantly favored the Prototype PTB Gumline mode over the Reference MTB (*p* < .0001) as well as the Prototype PTB Combined mode over the Prototype PTB Gumline mode (*p* < .0001), the Reference MTB (*p* < .0001), and the Reference PTB (*p* = .0010). The Reference PTB was significantly favored versus the Prototype PTB Gumline mode and the Reference MTB (both *p* < .0001).

#### 3.2.3 Proximal sites (D, E)

As with the gingival margin sites, prebrushing mean (±SE) score was the same for all toothbrush groups: 1.00 (±0.000). Also, similar, postbrushing scores reduced significantly for all toothbrushes (within group *p* < .0001) with the reduction being greatest for the Prototype PTB in Combined mode (Table 1; Figure 4). Between‐group comparisons (Table 2; Figure 4) of change from prebrushing scores significantly favored the Prototype PTB Gumline mode and the Reference PTB over the Reference MTB as well as the Prototype PTB Combined mode over the Prototype PTB Gumline mode and the Reference MTB (all *p* < .0001). There was no significant difference between the Prototype PTB in Combined mode versus the Reference PTB (*p* = .1652).

### 3.3 Safety evaluation

There were no training or treatment‐related AEs or any deaths or serious AEs during the study period.

## 4 DISCUSSION

The focus of this exploratory study was to investigate the ability of a novel Prototype PTB to remove dental plaque after a single brushing. A regular MTB and a Reference PTB were used for comparison. Maintenance of gingival health is a critical aspect of good oral health to prevent inflammation and the development of periodontal disease (Harvey, 2017). Toothbrushing is a vital part of oral health maintenance and is recommended by the European Federation of Periodontology as “a primary means of reducing plaque and gingivitis” (Sanz et al., 2020). To improve brushing techniques and compliance there are numerous different types of marketed PTBs available with different brushing actions such as moving from side to side or rotation‐oscillation (Ng et al., 2020). These can contribute to superior plaque removal in comparison to MTBs (Delaurenti et al., 2017; Ng et al., 2020; Yaacob et al., 2014).

In this study, reduction in the whole mouth, gingival margin, and proximal RMNPI scores postbrushing were significantly better following use of the Prototype PTB in Gumline and Combined (Gumline + Interdental brushing) modes compared to the Reference MTB. This reflects results reported previously where PTBs removed more dental plaque than an MTB after a single brushing (Klukowska, Grender, & Timm, 2012; Sharma et al., 2011; G. A. Van der Weijden et al., 1998). The Prototype PTB in Combined mode, though not Gumline mode, was also significantly better than the Reference PTB when assessing whole mouth and gingival margin plaque scores. As the Prototype PTB works in two different brushing axes, these results suggest that further efficacy in terms of plaque removal can be gained in a PTB by including this aspect of design. That both axes are needed is supported by findings that the Prototype PTB in Combined mode showed significantly better plaque removal efficacy than in Gumline mode for all RMNPI area scores.

PTBs are designed to increase compliance with features such as a 2‐min timer and 30 s quad pacers and, more recently, personalized apps to ensure that the user brushes correctly (Delaurenti et al., 2017; Erbe et al., 2018, 2019; Yaacob et al., 2014). However, while dental professionals recommend a toothbrushing routine of 2 min twice a day, in line with clinical trials (Sharma et al., 2011, 2012; G. A. Van der Weijden et al., 1993), observational studies find the estimated at home brushing time with an MTB is approximately 60 s or less (Emling et al., 1981; MacGregor, 1984; Nordstrom & Birkhed, 2017). Accordingly, in this exploratory study, a 1‐min brushing time for the MTB was used to represent the average duration of consumer brushing time with an MTB. This is reflected in several similar investigations where 1‐timed minute has been used (Conforti et al., 2003; G. A. Van der Weijden et al., 1998). In this study, compared with the MTB used for 1 min, percentage change from baseline was greater when the Prototype PTB was used for 2 min (Gumline mode) or for an additional 1 min (Combined mode) or when the Reference PTB was used for 2 min. It cannot be discounted that this difference was due to the longer brushing times; however, a previous study found that regardless of time spent brushing, the MTB always removed less plaque than a PTB. Differences may also be due to the two different toothbrush types used (manual and power brushes). G. A. Van der Weijden et al. (1993) Further studies could compare products using identical brushing times to ensure this was not the reason for differences between the MTB and the PTBs and only compare the Prototype PTB to other PTBs.

The design of this study, involving supervised single use, is in line with recommendations for initial assessment of the efficacy of a novel toothbrush and has been the methodology of choice in other studies (Biesbrock et al., 2007; Conforti et al., 2003; Klukowska, Grender, & Timm, 2012; Sharma et al., 2011; G. A. Van der Weijden, 2002; Williams et al., 2008). Further investigation could evaluate the effect of the Prototype PTB when used unsupervised in home settings over longer time periods (i.e., 3 months) to assess its longer‐term effect on oral health. Another study design to be considered is that while other studies have investigated plaque removal following longer periods of toothbrushing abstinence (Rosema et al., 2016), in a similar vein to retaining a 1 min brushing for the MTB, this study assessed plaque after 12 h as this would be similar to home practices. As the plaque reductions in this study were similar with both toothbrushes, a longer period of abstinence may show further differences between toothbrushes for future studies comparing PTBs.

No AEs or OST abnormalities were reported during the current study; hence, the products were deemed generally well tolerated. This is in line with numerous clinical studies that have evaluated the effect of PTBs on OST and there is a large body of evidence showing that oscillating‐rotating toothbrushes are considered as safe as using an MTB (F. A. Van der Weijden et al., 2011).

In conclusion, while the study was exploratory, it provided first evidence of a novel PTB design, which included a constant and low speed of rotation and a brush head that rotates in two different axes, providing two distinct cleaning modalities and significantly removing plaque in all areas of the teeth, particularly in the gingival margin.

## AUTHOR CONTRIBUTIONS

Paola Gomez‐Pereira, Alyson Axe, and Andrew Butler contributed to study design. Jimmy Qaqish and Chhaju Ram Goyal contributed to data acquisition. Andrew Butler contributed to data analysis. All authors contributed to the drafting of the manuscript and approved the final version.

## CONFLICTS OF INTEREST

This study was sponsored by GSK Consumer Healthcare, of whom P. G. P. and A. A. are employees and A. B. is a former employee. All Sum Research, of whom J. Q. and C. R. G. are employees, received funding for this study from GSK Consumer Healthcare.

## Data Availability

Anonymized individual participant data and study documents can be requested for further research from www.clinicalstudydatarequest.com.
